# Leaf anatomy of two reciprocally non-monophyletic mountain plants (*Heliosperma* spp.): does heritable adaptation to divergent growing sites accompany the onset of speciation?

**DOI:** 10.1007/s00709-016-1032-5

**Published:** 2016-10-07

**Authors:** Clara Bertel, Peter Schönswetter, Božo Frajman, Andreas Holzinger, Gilbert Neuner

**Affiliations:** Institute of Botany, University of Innsbruck, Innsbruck, Austria

**Keywords:** Adaptation, Alpine plants, Environmentally induced speciation, Leaf anatomy, Leaf ultrastructure

## Abstract

Evolution is driven by natural selection, favouring individuals adapted in phenotypic traits to the environmental conditions at their growing site. To shed light on ecological and (epi-) genetically based differentiation between *Heliosperma pusillum* and *Heliosperma veselskyi*, two reciprocally non-monophyletic, but morphologically and ecologically divergent species from the south-eastern Alps, we studied various leaf anatomical traits and investigated chloroplast ultrastructure in leaves of the two species grown either in their natural habitat or in a common garden. The alpine *H. pusillum* occurs in open, wet rock habitats, whereas its close relative *H. veselskyi* is restricted to dry, shady habitats below overhanging rocks in the montane belt. *H. pusillum* exhibited higher thickness of leaves and palisade layers as adjustments and/or adaptations to higher irradiance and a higher stomatal area index reflecting better water availability. Traits were adjusted plastically, but differed between species grown in a common garden, suggesting that the differentiation between the two species is not solely based on phenotypic plasticity but also has a genetic basis. Our study thus supports the hypothesis that differentiation between the highly interfertile species is likely driven by natural selection.

## Introduction

Adaptation to environmental conditions can become manifest in various phenotypic traits, as natural selection favours individuals being able to successfully survive and reproduce in a particular environment (Coyne and Orr 2004). Habitat heterogeneity, which is particularly pronounced in mountain areas (Scherrer and Körner 2011), may trigger the formation of ecotypes within species. Ecotypes originate from differentiation of populations that are adapted in various phenotypic traits to a specific microenvironment (Hufford and Mazer 2003; Lowry 2012). Ecotypic differentiation is most likely driven by a combination of heritable and non-heritable traits (Pfennig et al. 2010; Bonduriansky et al. 2012). Despite the absence of intrinsic reproductive barriers, ecological isolation may over time lead to the formation of new species (Lowry 2012).

**Fig. 1:**
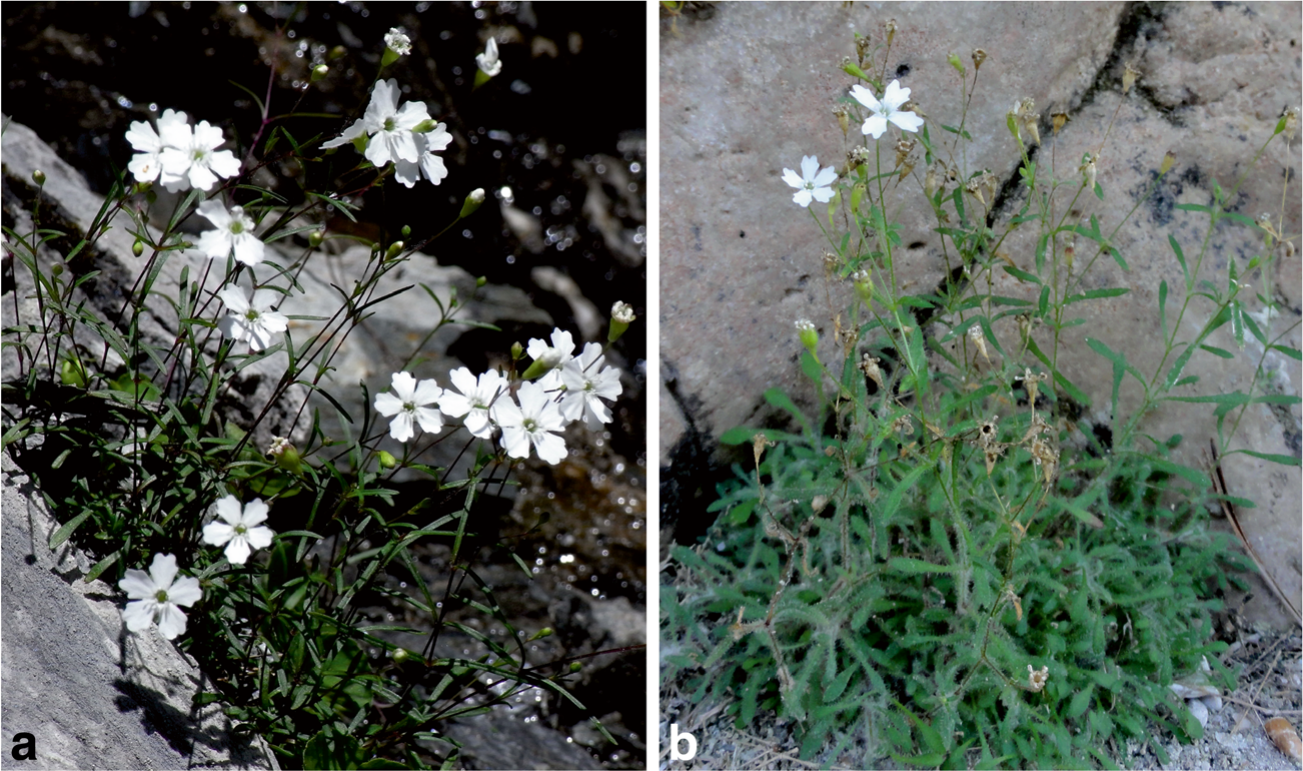
*H. pusillum* (**a**) grows on alpine scree sites, whereas *H. veselskyi* (**b**) occurs below cliff overhangs in the montane belt. *H. veselskyi* differs morphologically from its high elevation relative by broader leaves, which are covered by a thick indumentum of multicellular, glandular hairs. Despite their ecological and morphological divergence, both species are genetically inseparable (photos modified from Bertel et al. 2016; (**a**), R. Flatscher; (**b**), M. Sonnleitner)

An example of a recent ecotypic differentiation is provided by *Heliosperma pusillum* and *Heliosperma veselskyi* (Caryophyllaceae, Fig. 1). The former has a broad distribution throughout the southern and central European mountain ranges and occurs in humid, partly sun-exposed rock crevices and screes in the upper montane to alpine zone (1700–2300 m a. s. l.; authors’ personal observations). In contrast, *H. veselskyi* is restricted to a few scattered populations below cliff overhangs in the lower montane belt of the south-eastern Alps and northernmost Balkan Peninsula (500–1300 m a. s. l.; Neumayer 1923; Frajman and Oxelman 2007). Its growing sites are usually characterised by low irradiance and limited water availability (Bertel et al. 2016). Due to morphological divergence, both entities have been described at the species rank and are still treated as independent species (e.g. Fischer 2008; Frajman and Oxelman 2007; Poldini 2002)*. H. pusillum* is glabrous or has sparsely hairy leaves, whereas *H. veselskyi* is characterised by a dense indumentum of long multicellular glandular hairs (Janka 1858; Neumayer 1923; Frajman and Oxelman 2007; Fischer 2008). Chloroplast and nuclear low copy DNA sequence data (Frajman and Oxelman 2007; Frajman et al. 2009) as well as highly resolving restriction associated DNA markers sampled across the nuclear genome (Trucchi et al., unpublished) suggest that the two species are phylogenetically not distinct and that *H. veselskyi* is inextricably nested within *H. pusillum. H. veselskyi* thus rather represents a habitat specific ecotype, whose disjunct populations have evolved postglacially from geographically close populations of *H. pusillum*. For the sake of simplicity, we treat *H. pusillum* and *H. veselskyi* as species throughout the text in spite of the lack of consistent genetic divergence and their highly debatable taxonomic value.

Significant differences in environmental conditions between the habitats of *H. pusillum* and *H. veselskyi* comprise irradiation, temperature and soil water availability, and result in specific photosynthetic adjustments in both species (Bertel et al. 2016). In general, adjustments of photosynthesis are partly plastic, but also involve non-reversible traits influencing light absorption and carbon fixation at various organisational levels ranging from that of organelles within mesophyll cells over leaf anatomy up to the whole-plant level (Larcher 2003). Leaves of plants growing under low irradiance display a set of morphological, biochemical and physiological adjustments (Valladares and Niinemets 2008). They usually have a thinner mesophyll with no or only a few layers of palisade parenchyma with shorter cells and fewer chloroplasts per area with larger grana and more stromal thylakoids and fewer stomata per leaf surface area (Larcher 2003). Mesophyll conductance is often low contributing to a low overall photosynthetic capacity (Monti et al. 2009).

Low water availability often leads to the formation of more trichomes, highly waterproof cuticles and also influences stomatal density (Schulze et al. 2002). Fewer stomata on upper leaf surfaces prevent evapotranspiration, and more and shorter stomata on lower leaf surfaces allow a more precise regulation of gas exchange (Lösch 2001; Larcher 2003). As the formation of a particular leaf anatomy is both delimited by genetic constraints and the peculiar environmental growth conditions during development, investigations aiming at disentangling genetic and environmental effects of leaf structural traits ought to include plants grown at their natural growing sites as well as plants cultivated in a common garden under similar environmental conditions.

To shed light on ecological and (epi-)genetically based differentiation of leaf anatomy between *H. pusillum* and *H. veselskyi*, we address the following questions: (1) Does leaf anatomy differ between both species grown under the divergent ecological conditions of their natural habitats? A lack of differentiation would point to a weak ecological difference between habitats or minor relevance for the species, suggesting that plastic adjustment of physiological characteristics in the short term may be sufficient to compensate the divergent habitat conditions. (2) In order to resolve the extent of phenotypic plasticity vs. (epi-)genetically based determination of leaf anatomical traits, we further asked if leaf anatomy differed between the two species grown from seeds under similar environmental conditions in a common garden. Absence of genetic differentiation between the species is expected to lead to similar leaf anatomy under identical growth conditions, whereas intrinsic genetic constraints would limit the plastic adjustment of leaf anatomy. In addition, we investigated leaf ultrastructure of both species from their natural growing sites and from common garden qualitatively by transmission electron microscopy in order to evaluate subcellular rearrangements (e.g. abundance of grana stacks), which mirror ecological and (epi-)genetic differentiation.

## Materials and methods

### Study plants and experimental design

Leaf samples were both taken from plants in their natural habitat and from individuals grown from seeds in a common garden. Samples from natural growing sites originated from two geographically close and recently diverged (Trucchi et al., unpublished) populations of *H. pusillum* (Mt. Rudnigkofel, 46.76°N, 12.88°E, 2060 m a. s. l.) and *H. veselskyi* (Anetwände, 46.77°N, 12.90°E, 790 m a. s. l.) situated in the Lienzer Dolomiten, Austria. In autumn of 2013, seeds were collected from both populations and sown in pots filled with soil composed of compost, ground earth, lava, turf, quartz sand, rock flour and pumice gravel. Thirty individuals of each species were cultivated in a common garden in the Botanical Garden of the University of Innsbruck, Austria (47.27°N, 11.38°E, 600 m a. s. l.) under the same climatic conditions with adequate water supply. Environmental conditions differed between the natural growing sites of both species, as higher irradiance and temperature fluctuations were measured at the alpine site of *H. pusillum*. They also differed between the natural growing sites and the common garden, where high irradiance—comparable to the site of *H. pusillum*—and higher leaf temperatures—comparable to the site of *H. veselskyi—*were measured. Results of microclimatic measurements from the same experimental sites and the same vegetation period are presented in Bertel et al. (2016). Leaf samples were taken from adult plants. From here on, we use the following abbreviations for sample origin: HP_G_, *H. pusillum* grown in the common garden; HP_N_, *H. pusillum* collected in nature; HV_G_, *H. veselskyi* grown in the common garden; HV_N_, *H. veselskyi* collected in nature.

### Leaf anatomical traits

For investigation of leaf anatomical traits, fully developed stem leaves from middle parts of the upright shoots were collected randomly from 12 well-developed and randomly chosen individuals of each of the four groups. These leaves had developed in the collecting season and were in a physiologically active state, permitting comparison among groups. Cross sections were obtained from the middle part of the leaf lamina using a hand-microtome and investigated with a microscope (Olympus BX50, Olympus Optical Co., Tokyo, Japan) in combination with the software cellD (Version 3.1., Olympus Optical Co., Tokyo, Japan). Several leaf anatomical traits were measured on one cross section per individual, i.e. 12 cross sections from 12 individuals per study group: total thickness of leaves (LT), thickness of palisade parenchyma (PT), thickness of spongy mesophyll (SMT), thickness of cuticle on adaxial (CT_ad_) and abaxial (CT_ab_) epidermis, thickness of epidermis on adaxial (ET_ad_) and abaxial (ET_ab_) surfaces. Trait values represent the mean of three measurements per cross section. Relative proportion of palisade parenchyma (PT_rel_), spongy mesophyll (SMT_rel_) and intercellular spaces (IC_rel_) were estimated from five images per study group of leaf cross sections according to Kubinova (1993). Stomatal density and stomatal length on adaxial (SD_ad_, SL_ad_) and abaxial (SD_ab_, SL_ab_) leaf surface were measured from imprints. For imprints, nitrocellulose polish was applied on whole leaves as a thin film, and carefully removed after 5 min air drying with transparent adhesive tape, which was then placed on microscope slides. Stomatal density was determined from digital microscopic images of imprints as the mean number of stomata located within 30 randomly chosen grid cells of 100 × 100 μm in the middle section of leaves. Stomatal length was measured on digital images of imprints and is the mean of five measurements. Adaxial and abaxial stomata area indexes (SAI_ad_ and SAI_ab_) were calculated as the product of mean stomatal length and stomatal density (Ashton and Berlyn 1994; Gratani and Varone 2004; Gratani et al. 2014).

### Transmission electron microscopy

Stem leaves of the middle section of vertical shoots were sampled on sunny days. Samples of both species were taken at the same day at natural sites and in the common garden with smallest possible temporal delay (HP_N_ and HV_N_, 10.07.2013, between 13.00 and 15.30 h; HP_G_ and HV_G_, 30.06.2014 at 11.30 h). Leaf samples were immediately fixed (2.5 % glutaraldehyde in 50 mM sodium cacodylate buffer, pH 7.0) for 2 h and rinsed in the same buffer. Samples were postfixed in buffered 1 % OsO_4_ over night at 4 °C and further processed as described by Holzinger et al. (2007). Ultrathin sections were investigated in a Zeiss Libra 120 TEM at 80 kV.

### Statistical analyses

A principal component analysis (PCA) was applied to illustrate differentiation among the four study groups using the leaf anatomical traits LT, PT, SMT, ET_ad_, ET_ab_, CT, SAI_ad_ and SAI_ab_ standardised to zero mean and unit variance. As the Spearman correlation index was lower than 0.8 for any pair of characters, all characters were retained. Differences between study groups were compared by a one-factorial analysis of variance (ANOVA), conducted for each leaf anatomical trait separately. Significant differences between study groups were analysed by pairwise comparisons applying Bonferroni correction at a significance level of *α* = 0.05. Leaf anatomical traits grouped by species (thus pooling growing sites) were tested by a two-factorial ANOVA (*p* < 0.05); we only report but do not interpret the test comparing natural site and common garden pooling species as natural growing sites are strongly divergent. For all analyses, data transformations were conducted if normal distribution was not given: PT was transformed by common logarithm, IC_rel_ by natural logarithm and SL_ad_ to the power of four. For SMT, data were normally distributed, but errors were not homogenous; in this case Welch’s test and pairwise Wilcoxon rank sum test (for comparison of study groups) were applied. All statistical analyses were computed in R 2.14.0 (R Core Team 2014). PCA was calculated using the functions dudi.pca (package ade4: Dray and Dufour 2007) and the package plotrix (Lemon 2006) was used for graphical representations.

## Results

**Fig. 2:**
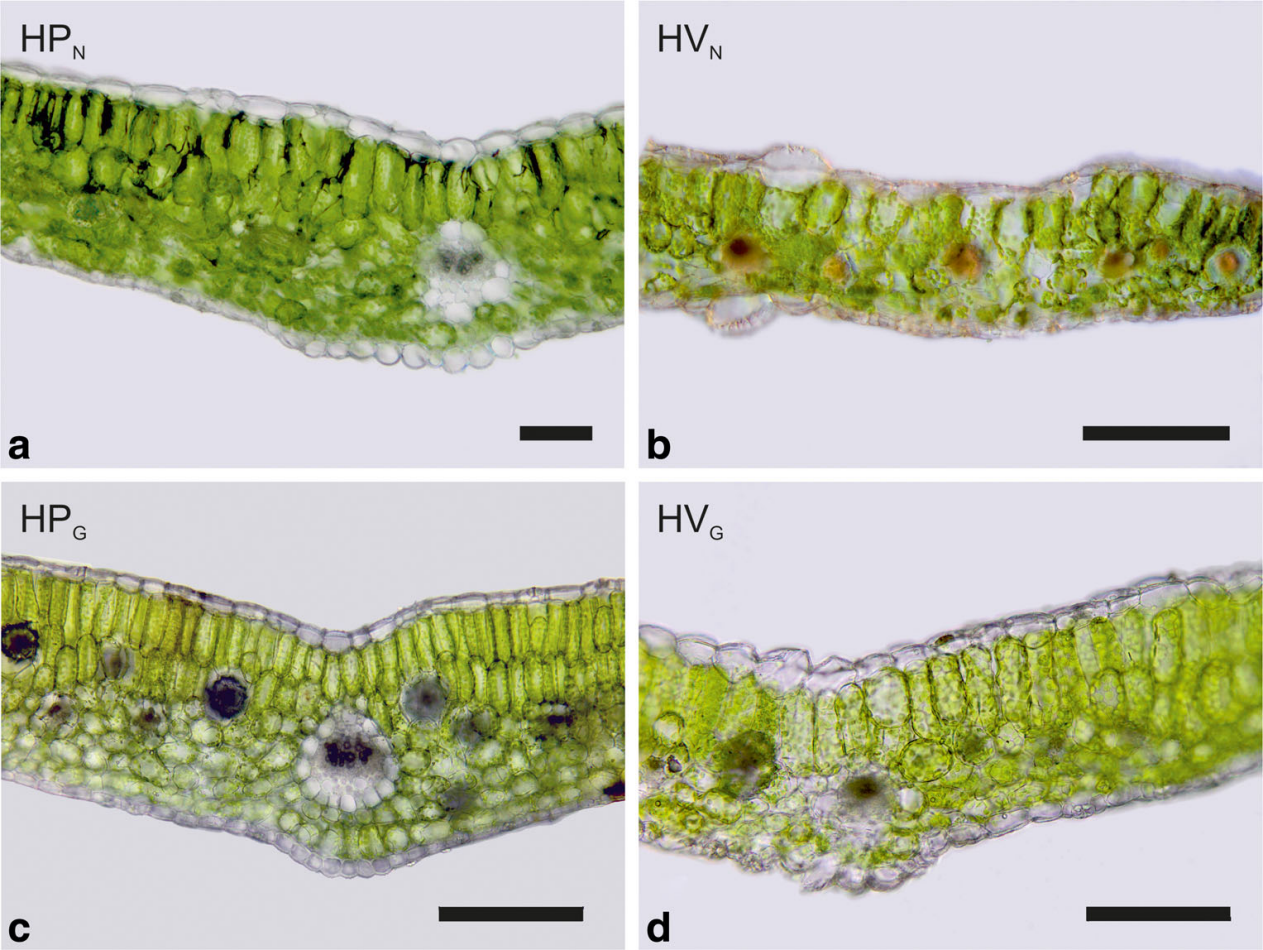
Light microscopy image of leaf cross sections of *H. pusillum* (**a**, **c**) and *H. veselskyi* (**b**, **d**) grown at their natural growing site (**a**, **b**) and in a common garden (**c**, **d**). *Scale bars*, 200 μm

**Fig. 3:**
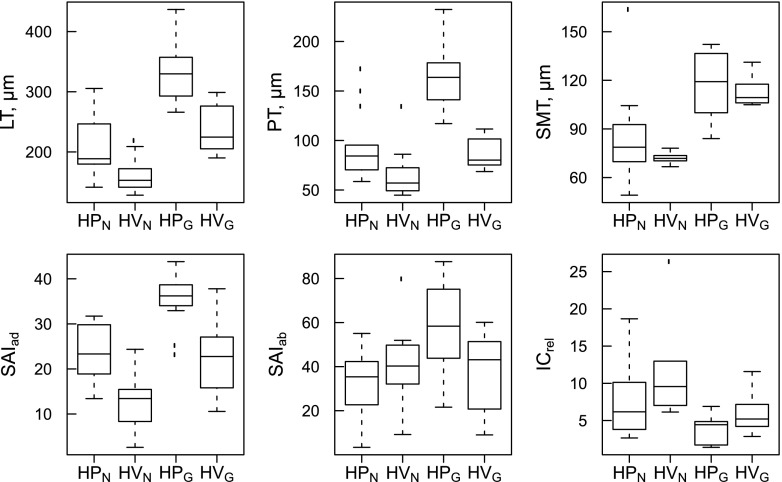
Leaf anatomical traits, i.e. total thickness of leaves (*LT*, *n* = 48), thickness of palisade parenchyma (*PT*, *n* = 48), thickness of spongy mesophyll (*SMT*, *n* = 48), stomatal area index on the adaxial (*SAI*_*ad*_, *n* = 39) and abaxial (*SAI*_*ab*_, *n* = 39) leaf surface and relative volume of intercellular spaces (*IC*_*rel*_, *n* = 21) determined on plants of *H. pusillum* and *H. veselskyi* grown at natural growing sites (HP_N_, HV_N_) and in a common garden (HP_G_, HV_G_). The box plots show the median, and the 25 and 75 percentiles. Values outside 1.5× interquartile ranges are labelled as outliers

Anatomical characteristics of leaves of *H. pusillum* and *H. veselskyi* from natural growing sites as compared to the common garden are illustrated in Fig. 2. Study groups differed significantly in most leaf anatomical traits according to the one-factorial ANOVA (Table 1). Differences in leaf anatomy were most pronounced in traits related to leaf thickness (i.e. LT, PT and SMT), with highest values in HP_G_, intermediate values in HP_N_ and HV_G_ and lowest values in HV_N_. In congruence, a second row of palisade parenchyma (No. P) was formed in all leaves of HP_G_ and in some leaves of HP_N_, but was never observed in HV_N_ or HV_G._ Further differences concerned thickness of cuticles (CT), epidermal layers (ET) and stomatal characteristics (SD, SAI, SL_ab_; boxplots for important traits are shown in Fig. 3, all traits are summarised in Table 1). Similarly, in the two-factorial ANOVA, the effect of species was significant for traits related to leaf thickness (LT, PT, PT_rel_) and surface characteristics (ETad, CT, SD_ad_, SAI_ad_, summarised in Table 2).

**Table 1:** Leaf anatomical traits (means ± SD) determined on plants of *H. pusillum* and *H. veselskyi* grown at the natural growing site (HP_N_, HV_N_) and in a common garden (HP_G_, HV_G_), as well as degrees of freedom (*df*) and *p* values obtained by one-way ANOVA

Leaf anatomical trait	HP_N_	HV_N_	HP_G_	HV_G_	*df*	*F*	*p*
LT (μm)	211.19 ± 50.91^a^	160.22 ± 29.17^b^	332.45 ± 47.96^c^	238.81 ± 39.49^a^	45	34.12	*<0.001*
No. P	1–2	1	2	1			–
PT (μm)	93.57 ± 36.28^a^	65.56 ± 25.07^b^	163.49 ± 31.98^c^	86.69 ± 15.85^a^	45	25.49	*<0.001*
SMT (μm)	84.38 ± 28.52^a^	71.98 ± 3.46^a^	116.55 ± 21.44^b^	112.56 ± 8.29^b^	45	101.51	*<0.001*
ET_ad_ (μm)	28.15 ± 4.68^ab^	28.23 ± 4.74^ab^	24.64 ± 4.01^a^	30.76 ± 5.98^b^	45	3.158	*0.035*
ET_ab_ (μm)	18.85 ± 3.17^ab^	15.92 ± 2.94^b^	19.52 ± 3.66^ab^	21.68 ± 4.97^a^	45	4.799	*0.005*
CT_ad_ (μm)	3.54 ± 0.86^ab^	3.05 ± 0.78^a^	4.17 ± 0.72^b^	3.53 ± 0.96^ab^	45	4.178	*0.011*
CT_ab_ (μm)	2.75 ± 0.49^a^	2.20 ± 0.49^c^	4.17 ± 0.67^b^	2.77 ± 0.50^a^	45	24.15	*<0.001*
SD_ad_	0.72 ± 0.23^a^	0.45 ± 0.23^a^	1.20 ± 0.28^b^	0.73 ± 0.32^a^	36	14.21	*<0.001*
SL_ad_ (μm)	32.27 ± 2.10	29.25 ± 5.06	29.82 ± 2.88	30.96 ± 2.16	45	1.439	0.242
SD_ab_	1.13 ± 0.25^a^	1.42 ± 0.54^a^	2.07 ± 0.53^b^	1.32 ± 0.34^a^	36	8.376	*<0.001*
SL_ab_ (μm)	35.38 ± 2.40^a^	31.61 ± 2.42^b^	30.82 ± 2.35^cb^	36.37 ± 4.26^a^	36	8.661	*<0.001*
SAI_ad_	23.20 ± 6.21^a^	12.73 ± 5.74^b^	35.39 ± 5.97^c^	22.81 ± 8.58^a^	36	15.92	*<0.001*
SAI_ab_	31.45 ± 17.96^a^	41.21 ± 17.33^ac^	56.60 ± 21.46^b^	37.48 ± 18.66^ab^	36	6.322	*0.014*
PT_rel_ (%)	37.88 ± 4.49	30.21 ± 3.41	38.91 ± 8.18	32.52 ± 7.80	18	2.634	0.081
SMT_rel_ (%)	32.14 ± 6.06^a^	37.84 ± 4.57^ab^	47.02 ± 7.02^b^	36.36 ± 4.97^ab^	18	6.432	*<0.001*
IC_rel_ (%)	7.93 ± 5.85^ab^	11.94 ± 7.52^a^	3.86 ± 2.30^b^	6.21 ± 3.39^ab^	18	3.317	*0.0434*

Traits include total thickness of leaves (LT), number of palisade cell layers (No. P), thickness of palisade parenchyma (PT), thickness of spongy mesophyll (SMT), thickness of epidermis on adaxial (ET_ad_) and abaxial (ET_ab_) leaf surface, thickness of cuticle on adaxial (CT_ad_) and abaxial (CT_ab_) epidermis, stomatal density, length and stomatal area index on adaxial (SD_ad_, SL_ad_, SAI_ad_) and abaxial (SD_ab_, SL_ab_, SAI_ab_) leaf surfaces, as well as relative proportions of palisade parenchyma (PT_rel_), spongy mesophyll (SMT_rel_) and intercellular spaces (IC_rel_). Differences between groups according to one-way ANOVA and multiple pairwise comparisons applying Bonferroni correction or pairwise Wilcoxon rank sum test (for SMT exhibiting unequal error variance) (*p* < 0.05) are indicated by different letters. Significant differences at *p* < 0.05 are indicated in italics

**Table 2:** Results of the two-factorial ANOVA

Leaf anatomical trait	*df*	MS_Residuals_	*F*	*p*
LT				
Species	1	59,798	32.456	*<0.001*
Site	1	123,236	66.888	*<0.001*
Species ***×*** site	1	5565	66.888	*0.089*
Residuals	45	1842		
PT (log10)				
Species	1	0.526	37.828	*<0.001*
Site	1	0.491	35.294	*<0.001*
Species ***×*** site	1	0.047	3.359	0.074
Residuals	45	0.0139		
ET_ad_				
Species	1	112.53	4.689	*0.038*
Site	1	3.63	0.151	0.699
Species ***×*** site	1	111.24	4.635	*0.037*
Residuals	45			
ET_ab_				
Species	1	1.71	0.121	0.730
Site	1	122.49	8.678	*0.005*
Species ***×*** site	1	79.04	5.6	*0.022*
Residuals	45	14.12		
CT_ad_				
Species	1	0.3361	6.570	*0.014*
Site	1	0.303	5.922	*0.019*
Species ***×*** site	1	0.0022	0.042	0.839
Residuals	45	0.0512		
CT_ab_				
Species	1	1.1917	33.044	*<0.001*
Site	1	1.3187	36.563	*<0.001*
Species ***×*** site	1	0.1022	2.834	0.099
Residuals	45	0.0361		
SD_ad_				
Species	1	1.456	20.768	*<0.001*
Site	1	1.440	20.528	*<0.001*
Species ***×*** site	1	0.092	1.317	0.259
Residuals	36	0.070		
SL_ad_				
Species	1	7.02 × 10^10^	0.594	0.446
Site	1	8.07 × 10^10^	0.683	0.414
Species ***×*** site	1	3.59 × 10^10^	3.039	0.090
Residuals	36	1.18 × 10^10^		
SD_ab_				
Species	1	0.1609	2.017	0.164
Site	1	0.7955	9.974	*0.003*
Species ***×*** site	1	1.0478	13.138	*0.001*
Residuals	36	0.0798		
SL_ab_				
Species	1	4.23	0.499	0.484
Site	1	0.06	0.007	0.932
Species ***×*** site	1	216.09	25.477	*0.001*
Residuals	36	8.48		
SAI_ad_ (^4)				
Species	1	1438.8	25.57	*0.001*
Site	1	1237.7	22	*0.001*
Species ***×*** site	1	10.1	0.18	0.674
Residuals	36	56.3		
SAI_ab_				
Species	1	401.3	2.543	0.120
Site	1	1770.8	11.22	*0.002*
Species ***×*** site	1	1082.6	6.86	*0.013*
Residuals	36	157.8		
PT_rel_				
Species	1	276.54	7.435	*0.014*
Site	1	15.16	0.408	0.531
Species ***×*** site	1	2.26	0.061	0.808
Residuals	18	37.19		
SMT_rel_				
Species	1	16.5	0.509	0.485
Site	1	244.7	7.543	*0.013*
Species ***×*** site	1	364.9	11.246	*0.004*
Residuals	18	584		
IC_rel_ (log)				
Species	1	1.413	3.691	0.071
Site	1	2.392	6.249	*0.022*
Species ***×*** site	1	0.004	0.010	0.922
Residuals	18	0.382		

The effect of species pooling growing sites (i.e. HP_N_, HP_G_ *vs.* HV_N_, HV_G_, “Species”), of growing site pooling species (i.e. HP_N_, HV_N_ *vs.* HP_G_, HV_G_, “Site”) and their interaction (“Species *×* Site”) were included as predictor variables for single leaf anatomical traits. As the natural growing sites of the two species are strongly divergent, the effect of growing site is not meaningful. Traits include total thickness of leaves (LT), thickness of palisade parenchyma (PT), thickness of spongy mesophyll (SMT), thickness of epidermis on adaxial (ET_ad_) and abaxial (ET_ab_) leaf surface, thickness of cuticle on adaxial (CT_ad_) and abaxial (CT_ab_) epidermis, stomatal frequency, length and stomatal area index on adaxial (SD_ad_, SL_ad_, SAI_ad_) and abaxial (SD_ab_, SL_ab_, SAI_ab_) leaf surfaces, as well as relative proportions of palisade parenchyma (PT_rel_), spongy mesophyll (SMT_rel_) and intercellular spaces (IC_rel_). Significant differences at *p* < 0.05 are indicated in italics. To achieve normal distribution and variance homogeneity, three variables were transformed by common (log10) or natural logarithm (log) or to the power of four (^4)

**Fig. 4:**
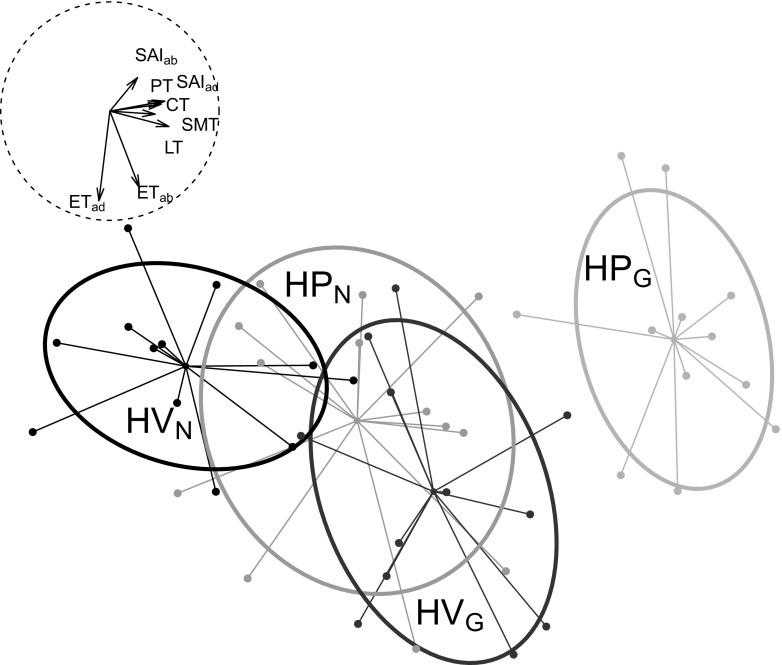
Leaf anatomical traits of *H. pusillum* (*dark grey*) and *H. veselskyi* (*light grey*) grown at their natural growing site (HP_N_, HV_N_) or in a common garden (HP_G_, HV_G_). Shown is a principal component analysis using the quantified leaf anatomical traits, i.e. total thickness of leaves (*LT*, *n* = 48), thickness of palisade parenchyma (*PT*, *n* = 48), thickness of spongy mesophyll (*SMT*, *n* = 48), mean thickness of cuticle on adaxial and abaxial epidermal layers (*CT*, *n* = 48), thickness of epidermis on adaxial (*ET*_*ad*_, *n* = 48) and abaxial (*ET*_*ab*_, *n* = 48) surfaces, and stomatal index on adaxial (*SAI*_*ad*_, *n* = 39) and abaxial (*SAI*_*ab*_, *n* = 39) leaf surface. Missing values of traits SAI_ad_ and SAI_ab_ were replaced by the group mean. Variables were transformed to achieve normal distribution, and scaled and centred. Confidence ellipses are defined by the centroid and the standard deviation of the cloud. Ordination axes represent 48.0 % (*x*-axis) and 17.2 % (*y*-axis) of the explained variance. *Arrows in the dashed circle* (*r* = 1) represent direction and magnitude of effects of structural traits

The PCA showed a clear differentiation between HP_N_ and HP_G_, HV_N_ and HV_G_ as well as between HP_G_ and HV_G_ (Fig. 4). The first three axes represented 76.2 % of variance (i.e. 47.9, 17.2 and 11.1 %, respectively). The traits PT, SAI_ad_, CT, SMT and LT correlated strongest with the first axis and SAI_ab_, ET_ab_ and ET_ad_ with the second axis.

**Fig. 5:**
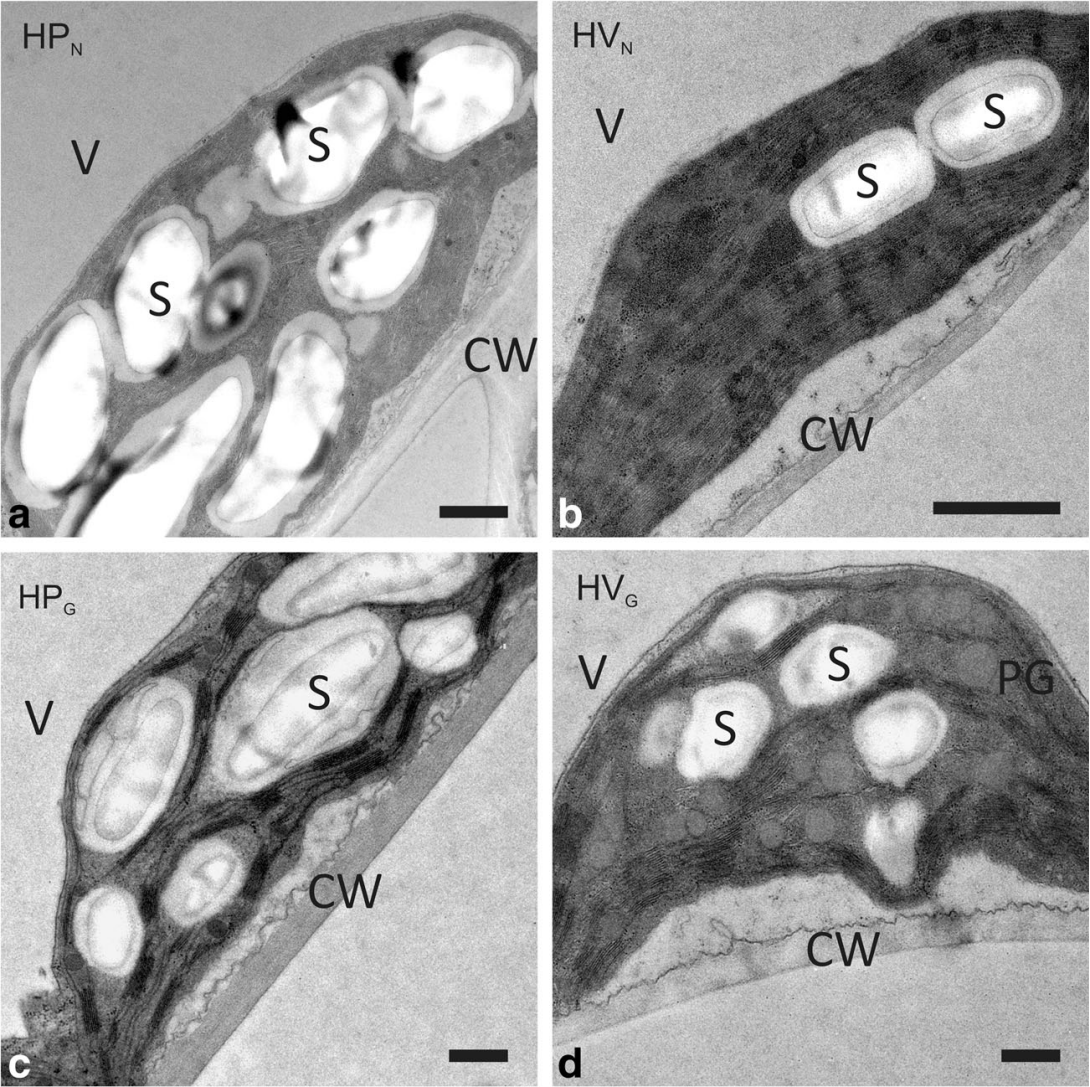
Transmission electron microscopy (TEM) image of chloroplasts of *H. pusillum* (**a**, **c**) and *H. veselskyi* (**b**, **f**) grown at their natural growing site (**a**, **b**) and in a common garden (**c**, **d**). Letters indicate starch grains (*S*), vacuoles (*V*), cell walls (*CW*) and plastoglobuli (*PG*); *scale bars*, 1 μm

The chloroplast ultrastructure is illustrated in Fig. 5. HP_N_ chloroplasts were more roundish-ellipsoidal and more densely packed with starch (Fig. 5a) than chloroplasts of HV_N_, which were lens-shaped containing only a few—mostly one or two—starch grains (Fig. 5b). Grana stacks contained more thylakoid membranes in HV_N_ than in HP_N_. Differences in chloroplast ultrastructure between HP_G_ and HV_G_ were less pronounced, but in HV_G_ plastoglobules were abundant in all examined chloroplasts, which were lacking or rarely observed in chloroplasts of all other study groups.

## Discussion

Leaf anatomy of the two mountain plant species *H. pusillum* and *H. veselskyi*, which occupy strongly divergent habitats despite their inextricable genetic relationship (Frajman and Oxelman 2007; Frajman et al. 2009), is differentiated both at their natural growing sites and in a common garden, suggesting that both species are heritably adapted to the particular conditions of their habitats in the Lienzer Dolomiten in Austria (Fig. 4). This is in line with evidence that populations of both species at this location have recently genetically diverged (Trucchi E, Frajman B, Haverkamp T, Schönswetter P and Paun O upubl.).

### Leaf anatomical traits reflect divergent environmental conditions

Environmental conditions at the natural growing sites of *H. pusillum* (HP_N_) and *H. veselskyi* (HV_N_) were reflected in their leaf anatomy, which was adapted to higher irradiance (PPFD) and better water availability in HP_N_. Thickness of leaves (LT) and palisade parenchyma (PT) were higher in HP_N_ than in HV_N_ (Fig. 3, Table 1), reflecting the higher irradiance at the growing site of HP_N_. Irradiance was characterised by an almost fourfold mean daily sum of photosynthetic photon flux density during the vegetation period at the HP_N_ site as compared with the HV_N_ site (Bertel et al. 2016). Increase in thickness of the palisade parenchyma—which contains the majority of chloroplasts and channels direct light, enabling plants to increase net photosynthetic rates (Boardman 1977; Björkman 1981; Vogelmann et al. 1996)—was due to longer cells and in some individuals to a second layer of palisade cells (Table 1). Congruently, higher net photosynthetic rates at high irradiance were measured in HP_N_ compared to HV_N_ (Bertel et al. 2016). Higher values of LT in HP_N_ could also be advantageous at its alpine growing site, as adjustment and/or adaptation to increased elevation often involves an increase in leaf thickness to compensate for the lower CO_2_ pressure at increased elevation (Körner 2003; Gratani et al. 2014).

LT was low in HV_N_ as was thickness of spongy mesophyll (SMT), but its relative proportion (SMT_rel_) and the relative proportion of intercellular spaces (IC_rel_) were in tendency higher in HV_N_ than in HP_N_ (Fig. 3, Table 1). This pattern is typically observed in shade-adapted leaves, as a relatively large proportion of spongy mesophyll enhances leaf absorbance due to greater internal light scattering at gas–liquid interfaces and thus returns photons to the palisade cells (Vogelmann et al. 1996). Intercellular spaces ranged from 4 to 12 % of the leaf cross section area and appear rather underestimated, as they usually comprise 30–40 % in dorsiventral leaves of other species (Vogelmann et al. 1996). Chloroplasts ultrastructure reflected the lower PPFD at the growing site of HV_N_, as chloroplasts were thinner and contained fewer starch globuli (Fig. 4). Thickness of grana thylakoids seemed to be elevated in HV_N_ as expected for shade-adapted leaves, since highly stacked grana enhance light absorption (Larcher 2003). However, the evidence remains scarce as our TEM analysis was only qualitative.

Adjustment and/or adaptation to drier growing sites is usually reflected in stomatal patterns (Larcher 2003). Under dry conditions, reduced stomatal density on upper leaf surfaces prevents evapotranspiration while more and shorter stomata on lower leaf surfaces allow a more precise regulation of gas exchange (Lösch 2001; Larcher 2003). The lower stomatal area index on adaxial leaf surfaces (SAI_ad_) measured in HV_N_ compared to HP_N_ may be advantageous at the drier growing site of HV_N_ as a lower SAI can limit transpiration (Lloyd and Woolhouse 1978; Dixon 1986; Gratani et al. 2014). It may also result from a lower photosynthetic demand for CO_2_ in HV_N_ as plants growing at low irradiance develop fewer stomata (Björkman 1981). However, on abaxial leaf surfaces, stomatal area index (SAI_ab_) did not differ between HV_N_ and HP_N_, and stomatal density (SD_ab_) was in tendency higher and stomatal length (SL_ab_) lower in HV_N_ (Fig. 3, Table 1). The higher stomatal density on adaxial leaf surfaces (SD_ad_) in HP_N_ may also be advantageous at higher elevation, as alpine plants often have more stomata and a higher proportion of stomata located on upper leaf vs. lower leaf surfaces. This most likely counteracts photosynthetic limitations resulting from lower CO_2_ pressure (Körner 2003; Aryal and Neuner 2012; Kammer et al. 2015). The reduced thickness of the cuticula (CT) observed in HV_N_ (Fig. 3, Table 1) is not necessarily directly related to an increased water permeability. The main barrier for water diffusion within the cuticula is located within a thin (1 μm) waxy band; hence, thickness may not reliably indicate overall water permeability (Kerstiens 1996; Lambers et al. 2008; Riederer and Schreiber 2001).

### Different leaf anatomical traits of *H. pusillum* and *H. veselskyi* grown in a common garden indicate an (epi-)genetically based differentiation between species

Leaf anatomy differed between *H. pusillum* and *H. veselskyi* when grown in the same common garden environment (HP_G_ and HV_G_; Figs. 2, 3, Tables 1, 2). Hence, the differentiation between the two species must be (epi-)genetically based to some extent. This is supported by the fact that in the common garden leaf anatomical traits (LT, PT, CT_ab_, SAI_ad_) were differentiated in the same direction between *H. pusillum* and *H. veselskyi* as at natural sites. Both species adjusted their leaf anatomy in response to the increased irradiance in the common garden, although the increase in irradiance was minor for *H. pusillum* but considerable for *H. veselskyi* (i.e. a 1.2- vs. 4.6-fold increase in daily sum of photon flux density; Bertel et al. 2016).

Adaptation to increased irradiance was mirrored in LT and PT, which was higher in HP_G_ than in HV_G_ (Table 1). The higher PT in HP_G_ was due to a second row of palisade cells, whereas in HV_G_, palisade parenchyma consisted of a single cell row. The number of rows of palisade cells might be genetically fixed as not all plants have the ability to build a second layer of palisade parenchyma (Lambers et al. 2008). In contrast, a similar SMT was attained in both species (Fig. 3, Table 1). We speculate that the different reaction norms for LT and PT reflect an adaptation to the natural growing sites, which are characterised by moderate to high irradiance at the site of *H. pusillum* vs. very low irradiance at the site of *H. veselskyi* (Bertel et al. 2016). The significant effect of species in the two-factorial ANOVA for leaf traits related to irradiance and water availability, such as LT, PT, PT_rel_, ET_ad_, CT_ad_, CT_ab_, SD_ad_ and SAI_ad_, indicates an (epi-)genetically based component of trait expression (Table 2).

Chloroplast ultrastructure is known to be highly plastic (Larcher 2003) and did not differ remarkably between the species. However, indications for adjustments and/or adaptations to shade in HV_N_ are given by the larger grana stacks. The considerable amount of plastoglobules in HV_G_ may indicate some sort of irradiation stress for *H. veselskyi* compared to *H. pusillum*, as the formation of plastoglobules usually results from the degradation of thylakoid membranes (Austin et al. 2006). Similar observations were quantitatively analysed in drought-stressed *Picea* plants, where the chloroplast volume occupied by thylakoid membranes increased from 27 to 37 %, and that of plastoglobules from 2 to 12 %, respectively (Zellnig et al. 2010). We are aware that such changes are reversible; however, they are a good indication of the physiological plasticity that will occur under continuously altered environmental factors.

In conclusion, our study, even if based on the comparison of a single population each of both investigated species, supports the hypothesis that differentiation between the two postglacially diverged species *H. pusillum* and *H. veselskyi* (Trucchi et al., unpublished) is likely driven by natural selection as (1) differentiation in leaf traits at natural growing sites was in congruence with environmental habitat conditions and (2) differentiation in the common garden reflected adaptation to the conditions of their growing site. Continuing studies investigating phenotypic differentiation among six population pairs and the fitness of each species in the habitat of the other will further contribute to our understanding of the functional differentiation between *H. pusillum* and its multiply independently evolved, rare and ecologically peculiar descendant *H. veselskyi*.
